# Radiofrequency Heating of the Cornea: An Engineering Review of Electrodes and Applicators

**DOI:** 10.2174/1874120700701010071

**Published:** 2007-12-11

**Authors:** Enrique J Berjano, Enrique Navarro, Vicente Ribera, Javier Gorris, Jorge L Alió

**Affiliations:** 1 Institute for Research and Innovation on Bioengineering, Technical University of Valencia, Valencia, Spain; 2 Neptury Technologies, Almassora, Spain; 3 Cornea and Refractive Surgery Department, Vissum-Instituto Oftalmológico de Alicante, Alicante, Spain; 4 Pathology and Surgery Department, Universidad Miguel Hernández, Elche, Spain

## Abstract

This paper reviews the different applicators and electrodes employed to create localized heating in the cornea by means of the application of radiofrequency (RF) currents. Thermokeratoplasty (TKP) is probably the best known of these techniques and is based on the principle that heating corneal tissue (particularly the central part of the corneal tissue, i.e. the central stroma) causes collagen to shrink, and hence changes the corneal curvature. Firstly, we point out that TKP techniques are a complex challenge from the engineering point of view, due to the fact that it is necessary to create very localized heating in a precise location (central stroma), within a narrow temperature range (from 58 to 76ºC). Secondly, we describe the different applicator designs (i.e. RF electrodes) proposed and tested to date. This review is planned from a technical point of view, i.e. the technical developments are classified and described taking into consideration technical criteria, such as energy delivery mode (monopolar versus bipolar), thermal conditions (dry versus cooled electrodes), lesion pattern (focal versus circular lesions), and application placement (surface versus intrastromal).

## INTRODUCTION

**Fig. (1):**
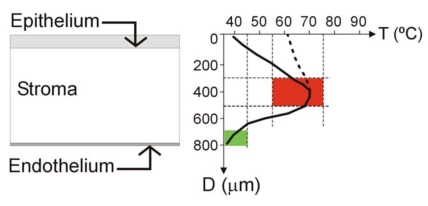
Left: Schematic diagram of the cornea showing the anterior layer (epithelium), the posterior layer (endothelium), separated by the stroma. The aim of the thermokeratoplasty (TKP) procedure is to create safe localized heating in the central stroma. Right: Optimum (solid line) and sub optimum (dotted line) thermal profiles in the cornea for themokeratoplasty (TKP). TKP techniques are a complex challenge from the engineering point of view, due to requiring: 1) an extremely high spatial resolution (i.e. it is necessary to create very localized heating in a precise location of the central stroma –red zone–, thermally protecting the endothelium –green zone–, which is placed no more than 300 μm distant from central stroma); and 2) a high resolution of the temperature reached at the target point (temperatures ranging from 58 to 76°C are required to shrink the collagen, and temperatures over 79°C are harmful). Since the epithelium is a layer with regeneration capability, it can stand heating during TKP(dotted line). However, in the optimum TKP technique the epithelium should be kept cool (solid line) and the heat focused on the central stroma.

Localized heating of the cornea has been employed since 1889 for different therapeutic and surgical objectives. The best known of these techniques is probably thermokeratoplasty (TKP), which is based on the principle that heating corneal tissue (particularly the central part of the corneal tissue, i.e. the central stroma) causes collagen to shrink, and hence changes the corneal curvature [1]. However, the process can be used for other therapeutic objectives. TKP is a complex challenge from the engineering point of view (see Fig. **1**). On one hand, it is necessary to create very localized heating in a precise location (central stroma), keeping the endothelium thermally protected (which is located no more than 300 μm distant from the central stroma). The endothelium is a mono-cellular layer in the human eye which is non regenerable, and hence the temperature at this point should always be maintained at a safe level (e.g. lower than 45ºC). Consequently, the procedure requires an extremely high spatial resolution. The temperature at the epithelium is not so critical: some TKP techniques combine surface cooling to create a temperature profile with a low temperature at the epithelium (see solid line in Fig. **1**). In contrast, other techniques (like intrastromal applicators) heat both the epithelium and the central stroma (see dashed line in Fig. **1**).

It is known that collagen shrinkage occurs at temperatures ranging from 58 to 76ºC. However, higher thermal levels, 79ºC or over, definitely lead to relaxation of the collagen and complete loss of its elasticity, inducing important keratocyte proliferation and accelerating collagen turnover (i.e. provoking a regression of the corrected refractive error) [2]. Therefore, the procedure also requires high resolution of the temperature reached at the target point (see Fig. **1**).

In order to create corneal heating, different kinds of energy sources have been tested, such as simple thermal conduction from pre-heated probes, known as thermokeratophores [3,4], microwaves [5-7], laser [2] and ultrasound [8-10], and radiofrequency (RF) currents. However, our interest is focused on techniques for corneal heating by means of RF currents (≈500 kHz), therefore in this review we describe the different designs of applicators (i.e. RF electrodes) proposed and tested to date. This review is planned from a technical point of view, i.e. the technical developments are classified and described taking into consideration technical criteria, such as energy delivery mode (monopolar versus bipolar), thermal conditions (dry versus cooled electrodes), lesion pattern (focal versus circular lesions), and application placement (surface versus intrastromal). Consequently, the developments are not necessarily related to either clinical procedures or trademarks.

## APPLICATORS WITH SURFACE COOLING

**Fig. (2):**
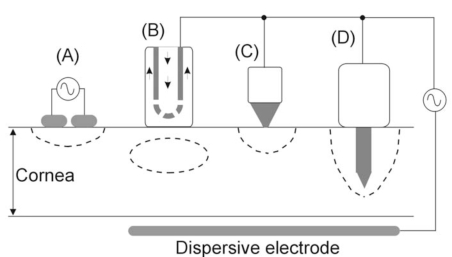
RF electrodes and applicators for creating a lesion at a certain point in the cornea. **A**: A pair of electrodes cause a lesion by means of a bipolar application. **B**: The circulating saline electrode (CSE) works by delivering RF currents from the active electrode to a dispersive electrode while a flow of isotonic saline is infused over the cornea surface. **C**: A small surface electrode is placed on the cornea and RF currents are applied between this and a dispersive electrode. The lesion is hence confined to the cornea surface. **D**: An intrastromal electrode is a needle-shaped electrode which penetrates from the surface to the central stroma. The lesion has more depth than in the case of a surface electrode (C).

The first applicator designed for applying RF currents in the human cornea was described by Doss and Albillar [11] in 1980. Since it was developed at Los Alamos Scientific Laboratory (NM, USA), the device was named the *Los Alamos Keratoplasty probe* [1]. However, Doss and Albillar called it the *circulating saline electrode* (CSE), since, while the active electrode delivered RF currents (1.6 MHz) a flow of isotonic saline (at 37ºC) was infused over the cornea surface [11,12] (see Fig. **2B**).

The basic idea of the CSE was to improve the temperature profile obtained by the thermokeratophore during thermokeratoplasty (TKP). The thermokeratophore was a metallic probe preheated to a specific temperature range (90-130ºC) and placed on the cornea surface, i.e. the thermal lesion was created by thermal conduction from the thermokeratophore towards the corneal stroma. Consequently, the maximum temperature was reached at the cornea surface (≈75ºC), while the central stroma remained at ≈45ºC [11]. In contrast, using the CSE, the maximum temperature in the cornea was reached at the central stroma (≈70ºC), both epithelium and endothelium remaining at a temperature lower than 50ºC. The applicator had a monopolar system, i.e. the RF currents were delivered between the CSE (active electrode) and a large dispersive electrode placed away from the CSE. This technique was first tested on excised pigs’ eyes [11], later on an in vivo model with dogs’ eyes (sacrificed within minutes of the treatment) [13], and finally some preliminary clinical trial were conducted on patients to treat keratoconus [14-16]. Although the CSE was very effective right away (the corneas were flattened), it was also a common experience that the flattening of these diseased corneas tended to diminish significantly within a few weeks. Unfortunately, the protocol did not allow performing re-treatments to determine whether longer term stability could be achieved (Doss, personal communication, Oct. 11, 1995). It is also important to point out that this technique has never been employed on relatively “normal” human corneas such as those found in astigmatism and hyperopic conditions (in contrast, in keratoconus the cornea has abnormal morphology and is also very thin).

The CSE technique was also proposed using a bipolar system (i.e. without using a dispersive electrode). Several arrangements with a variable number of electrodes were proposed [17] but never tested. The most novel idea of CSE was to cool the cornea surface during heating. In fact, the same idea was later employed in microwave thermal keratoplasty [5-7], and more recently, a system for surface cooling of the cornea during TKP has been patented, especially for electrical-induced techniques such as RF or microwave TKP [18].

## APPLICATORS WITHOUT SURFACE COOLING (DRY APPLICATORS)

Prior to the development of the CSE, some experimental studies were conducted by using a two-electrode applicator for delivering RF currents (2 MHz) in a bipolar system (see Fig. **2A**). The applicator, known as the* localized current field (LCF) device*, was also developed at Los Alamos Scientific Laboratory [19]. It was employed in the veterinary field for hyperthermic therapy for neoplasia or degenerative corneal diseases [20,21]. A similar probe, i.e. based on a bipolar RF application, was later proposed for refractive surgery [22]. It consisted of a bipolar forceps which created thermal lesions in segments of perilimbal corneal tissue. To our knowledge it has never been tested.

Around 1993, Mendez and Mendez-Noble [23] proposed using a small surface application electrode to create small lesion spots (see Fig. **2C**). The aim was the correction of hyperopia by producing steeping of the cornea by means of creating a series of eight spots of one millimeter in diameter distributed symmetrically around a seven millimeter diameter ring in the mid-periphery of the cornea. The electrode played the role of an active electrode, and a large-area disk was the dispersive electrode. In addition, a more complex applicator including up to eight electrodes was proposed to simultaneously create the circular lesion pattern [24]. Our group also conducted computational and experimental research to characterize the corneal lesions created by this kind of surface electrode application [25,26]. In a retrospective study of 166 cases Mendez and Mendez-Noble [23] found some cases of regression due to surface application. They considered that during the surface application, tear film or balanced salt solution on the cornea might cause a dissipation of the energy. In 1994 they therefore proposed the intrastromal application of RF energy by means of penetrating electrodes (see subsequent section).

**Fig. (3):**
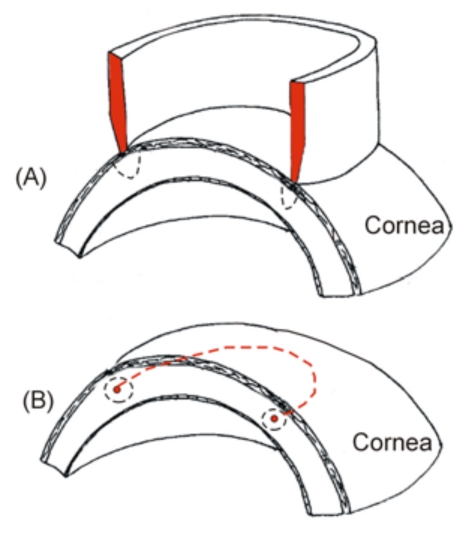
Two types of RF electrodes for creating a circular lesion in the cornea (both use a monopolar system, i.e. the RF current circulates between the electrode and a dispersive electrode, which is not shown). A: A circular electrode placed on the cornea provides a circular lesion pattern. B: A small circular electrode is inserted into the central stroma (red dashed line), providing a toroidal lesion.

Finally, we also conducted experimental research to assess the performance of a ring applicator for the rapid creation of a circular lesion pattern [27] (see Fig. **3A**). The results suggested that the contact conditions between applicator and cornea surface should be balanced along the circular path in order to avoid zones with different lesion characteristics. In fact, it has been recognized that manual placement of the probe may involve human error, and hence the problem of controlling the contact between applicator and cornea has been broadly investigated in RF heating [28-30].

## INTRASTROMAL APPLICATORS

The electrodes most frequently employed in clinical practice for RF heating of the cornea are the intrastromal electrodes. They were initially proposed by Mendez and Mendez-Noble in 1994 in order to improve the results obtained by surface application electrodes [23]. Briefly, the active electrode has a very fine tip that measures between 350 and 400 μm in length and ≈100 μm in diameter. It is inserted into the cornea and then the RF energy is directly applied into the stroma. Typical values are a frequency of 330-350 kHz [31-33], and a power of ≈600 mW delivered for ≈600 ms.

**Fig. (4):**
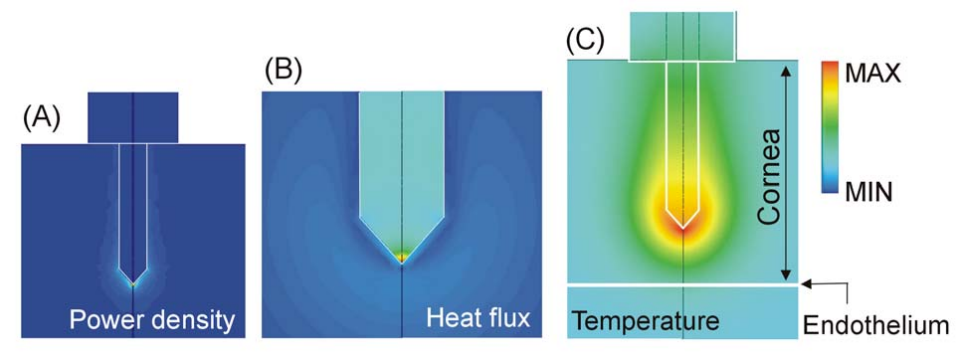
Computer modeling of an intrastromal electrode. This kind of electrode has two important advantages: 1) the RF power is mainly deposited at the electrode tip (see Joule heat in **A**), and 2) the low thermal conductivity of the electrode metallic body facilitates the transmission of heat from the electrode tip to the anterior zone (see heat flux in **B**). Note that the maximum heat flux is located in the electrode, and in contrast, the corneal tissue shows a very low heat flux value. A high thermal gradient therefore appears between the electrode tip and endothelium, which provides thermal protection to the endothelium (see temperature in **C**). Scales are normalized.

The electrical-thermal behavior of this kind of electrode has been extensively studied using computer modeling [34-38]. The same kind of electrode has also been employed in other RF heating techniques such as ablation of cardiac arrhythmias [39]. It is known that RF power is mainly deposited at the electrode tip (see Fig. **4A**). This behavior is especially advantageous for heating the cornea, since it allows direct heating of the central stroma. However, this electrode design offers additional advantages for cornea heating; since the electrode body is usually made of a conducting metal (e.g. stainless steel), the low thermal conductivity of this element compared to the thermal conductivity of the cornea facilitates the transmission of heat from the electrode tip to the anterior cornea (see Fig. **4B**). As a result, a high thermal gradient appears between the electrode tip and endothelium, which provides thermal protection to the endothelium (see Fig. **4C**).

**Fig. (5):**
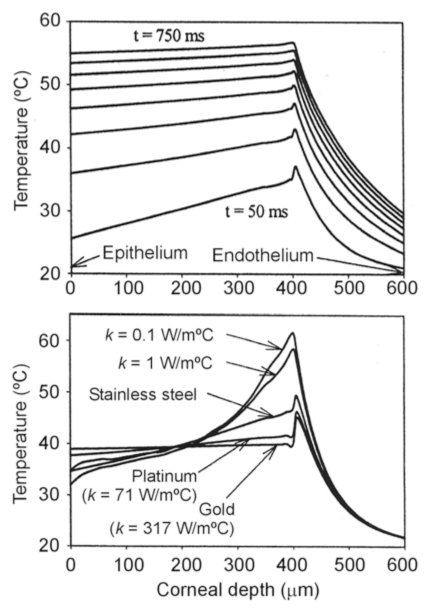
Computer modeling of intrastromal electrodes. Top: Progress of the temperature profiles (assessed on the electrode axis) for an intrastromal electrode made of stainless steel (each plot represents a time step of 10 ms). Bottom: Temperature profiles at 100 ms for intrastromal electrodes with different thermal conductivity values. Note that electrodes with low thermal conductivity can remove the heat generated at the electrode tip more efficiently. All the simulations were conducted by applying a constant voltage of 10 V and the electrode tip was located at a depth of 450 µm in the cornea [41].

We studied the effect of the thermal conductivity of the intrastromal electrode on the temperature profile in the cornea by means of theoretical modeling [40]. Fig. **5** shows the temperature profiles for intrastromal electrodes made from different materials, confirming the importance of the electrode body in diffusing the heat created at the electrode tip.

Finally, regarding intrastromal electrodes, it is interesting to note Silvestrini’s proposal [41] for an RF applicator circular in shape (or semicircular or any other appropriate form) which is placed on the central stroma and inserted into the inner cornea through at least one access (see Fig. **3B**). To our knowledge this device was never experimentally assessed.

## COMPUTER MODELING STUDIES

During the study and development of applicators for RF heating of the cornea, theoretical models and computer simulations have played an important role. This methodology is advantageous, being both fast and cheap, for assessing the electrical and thermal behavior of RF applicators [42]. For instance, computer modeling studies have been developed for surface applied monopolar electrodes, both for single [25] and circular lesion patterns [27]. Likewise, many computer modeling studies have been carried out on intrastromal electrodes, especially for those employed in Conductive Keratoplasty (CK) [34-38].

## CLINICAL RELEVANCE

Even though numerous RF applicator designs for cornea heating have been proposed and experimentally tested, to date only the needle-shaped intrastromal applicators have achieved clinical success [31,32,43]. For instance, they were initially employed for the correction of low to moderate hyperopia [44], and to correct residual hyperopia after corneal surgery [45]. Subsequently, clinical trials were carried out on correcting hyperopic astigmatism [46] and presbyopia [47]. Corneal modeling of keratoconus has recently been conducted by means of this type of electrode [43].

From the clinical and manufacturing point of view, these electrodes were initially manufactured by Refractec (Irvine, CA, USA). This company currently develops and markets electrodes and an RF generator under the trade name of ViewPoint® CK System. The surgical procedure is known as Conductive Keratoplasty® (CK). In general, for the treatment of hyperopia, the lesion spots (which shrink corneal collagen in the treatment zone) are created following a full circular pattern (6, 7 and/or 8 mm diameters). This lesion pattern acts like a belt, tightening the peripheral region of the cornea so that the central cornea becomes slightly raised [48].

The Brazilian company Loktal Medical Electronics (Butantã, São Paulo, Brazil) has also developed similar electrodes (stainless steel tip of 90 µm in diameter and 350 µm in length) which are employed with an RF generator (Wavetronic Genius). To date, this equipment has been employed in a clinical study for treatment of advanced keratoconus [32]. In this case, the thermal spots were created in optical zones of 4.0 and 5.0 mm (the exact number of spots and their location were determined by the degree of corneal curvature) [32].

Both technologies (Refractec and Loktal) are based on delivering RF energy for a total time of 600 ms (typical value). During this period, the RF generator emits a train of pulses consisting of exponentially damped sinusoidal waves (350 kHz) [31,32,38]. The typical value of delivered power is 600 mW, however other power values can be employed. On this topic, Choi et al [33] have studied in depth the effect of programmed power on impedance progress during heating.

## CONCLUSION

This paper has reviewed the different applicators and electrodes employed to create localized heating in the cornea by means of the application of radiofrequency (RF) currents. Taking into consideration the extensive number of cornea heating techniques for purposes of refractive surgery proposed and tested to date (thermokeratophores, laser, microwave, ultrasound, radiofrequency), we believe that the problem has not yet been solved. Therefore, a great deal of research effort should be focused on finding the optimum RF applicator for cornea heating.
